# Clinical results with a multifocal intraocular lens with a novel optical design

**DOI:** 10.1186/s12886-024-03521-7

**Published:** 2024-06-26

**Authors:** Gustavo Goldman

**Affiliations:** Ophthalmological practice Dr. Goldman, Teodoro Garcia 2099, Piso 2, Buenos Aires, 1426 Argentina

**Keywords:** Cataract, Dysphotopsia, Endothelial cell density (ECD), Intraocular lens (IOL), Presbyopia

## Abstract

**Background:**

To evaluate the optical performance and safety of a new multifocal lens with a novel optical design featuring two additional foci (or intensifiers) in patients with cataract and presbyopia.

**Methods:**

In this single-center, non-randomized prospective observational study, 31 patients underwent implantation of the new multifocal IOL between March 2020 and November 2021 at a tertiary clinical center in Buenos Aires and Ramos Mejia, Argentina. Postoperative examinations with emphasis on uncorrected and corrected visual acuity at distance and near and at two different intermediate distances (80 cm and 60 cm) were performed during the 3 postoperative months.

**Results:**

Of the 31 patients who underwent implantation of the new IOL, 30 underwent bilateral surgery (61 eyes in total). At 3 months, all 61 eyes had an uncorrected distance visual acuity (UCDVA) of at least 0.15 logMAR; 57 eyes (93%) had an uncorrected distance visual acuity (UCDVA) of 0.1 logMAR and 27 eyes (44%) had an UCDVA of 0.0 logMAR. At 80 cm, 60 eyes (98%) had an uncorrected intermediate visual acuity (UCIVA) of at least 0.1 log MAR and 48 eyes (79%) had an UCIVA of 0.0 logMAR.

**Conclusion:**

The new multifocal IOL with a novel optical concept (5 foci) showed a wide range of visual acuity especially at intermediate and near distances in patients undergoing cataract surgery. Uncorrected visual acuity was excellent at all tested distances, monocularly and binocularly, spectacle independence and patient satisfaction were high.

**Supplementary Information:**

The online version contains supplementary material available at 10.1186/s12886-024-03521-7.

## Background

Cataract surgery with implantation of an intraocular lens (IOL) is one of the most frequently performed procedures in modern medicine worldwide. Since cataract is generally a condition that affects people in the second half of their lives, presbyopia is a common comorbidity or, if that term sounds too strong, a common associated refractive status. In fact, presbyopia is the most common refractive disorder in people over the age of 40 [1].

The generation of patients approaching or already at the age when cataract surgery becomes necessary is generally well educated and comes to the cataract surgeon with certain preconceptions and expectations. These patients often have active lifestyles beyond retirement, or sometimes have no intention of retiring. One of their expectations is to be able to continue this lifestyle after cataract surgery with less or even no need to wear glasses [2]. An essential part of these changing lifestyles in our time is the rise of digital devices such as laptops, tablets and smartphones and their widespread use, even by older people. In particular, the intermediate distance of about 60 cm (about 23 inches) plays an essential role in a daily life dominated by electronic devices.

In recent years, many new IOLs have been introduced; advanced and/or optical designs have recently entered the market with encouraging initial clinical results [3] To provide good visual acuity over a range of distances and to keep patients “spectacle free”, a number of extended depth of focus (EDOF) lenses as well as multifocal IOLs have been developed. While these devices improve intermediate and near vision (although usually less than perfect), multifocal lenses in particular are associated with visual disturbances such as halos and glare [4] and a higher incidence of dysphotopsia and poorer contrast sensitivity have been reported in patient-centered outcomes with existing multifocal IOL designs [5].

The Intensity SL is a new multifocal IOL based on a novel optical design. According to the manufacturer (Hanita Lenses, Israel), a process called Dynamic Light Utilization, based on a proprietary algorithm developed by the company, is used to give this lens a specific profile that allows continuous and supposedly uninterrupted vision throughout the entire visual field. The optical profile of the lens consists of 12 steps with a central zone of 1 mm diameter. In this prospective, non-randomized, single-arm, single-center observational study, we evaluated the optical efficacy of the Intensity SL in a real-world clinical setting.

## Methods

### Study design

This single-center, non-randomized, prospective, observational study was designed to evaluate the efficacy and safety of the Intensity SL posterior chamber IOL in cataract surgery and presbyopia correction. Thirty-one patients provided informed written consent to participate in the study. Surgery was performed between March 2020 and November 2021 with a 3-month follow-up after surgery on the second eye. The surgeries were performed with a one-week interval between the two eyes. Preoperatively, the IOL power was calculated with the IOL Master using the SRKT formulas. Postoperative examinations were performed on the first day after surgery, at 1 month, 2 months and 3 months. A complete examination included visual acuity measurement with Snellen charts, visual acuity was divided into binocular and monocular vision. Uncorrected visual acuity (UCVA) was assessed for distance (UCDVA), intermediate (UCIVA) and near (UCNVA) vision under photopic and mesopic conditions. In addition, best (distance) corrected visual acuity (BCVA) was assessed for distance (BCDVA), intermediate (BCIVA), and near (BCNVA) vision. Intraocular pressure (IOP) was measured with the Goldmann tonometer, and endothelial cell density (ECD) was measured with the Konan cellcheck. Slit-lamp examination with focus on signs of anterior chamber inflammation and assessment of IOL tilt, decentration or other device defects and fundus examination were mandatory at each follow-up visit.

The study was approved by the local ethics committee (Comité de Ética, Consejo Argentino de Ofthalmología) and was conducted in accordance with the tenets of the Declaration of Helsinki.

### Eligibility criteria

Inclusion criteria were age 45 years and older, bilateral age-related cataract requiring cataract surgery. The cornea had to be normal with an astigmatism not exceeding 0.75 diopters (D). The fundus had to be visualized without major posterior segment pathology. Patients had to be motivated to receive an Intensity SL after being thoroughly informed by the surgeon about the procedure and the characteristics of this IOL design. Preoperative endothelial cell count had to be greater than 2000/mm^2^.

Exclusion criteria were previous ocular and especially corneal surgery (such as corneal refractive surgery) that could have affected visual and refractive outcomes. Other exclusion criteria were pupil abnormalities such as inability to dilate at least 3.5 mm under mesopic/scotopic conditions, amblyopia, glaucoma, and any other ocular disease that could affect visual acuity (patients with vitreous floaters could be included). Specific and rare forms of cataract such as traumatic cataract and rubella cataract were excluded, as were capsule and zonular abnormalities such as pseudoexfoliation syndrome, Marfan syndrome, and chronic uveitis.

### IOL device description

**Fig. 1:**
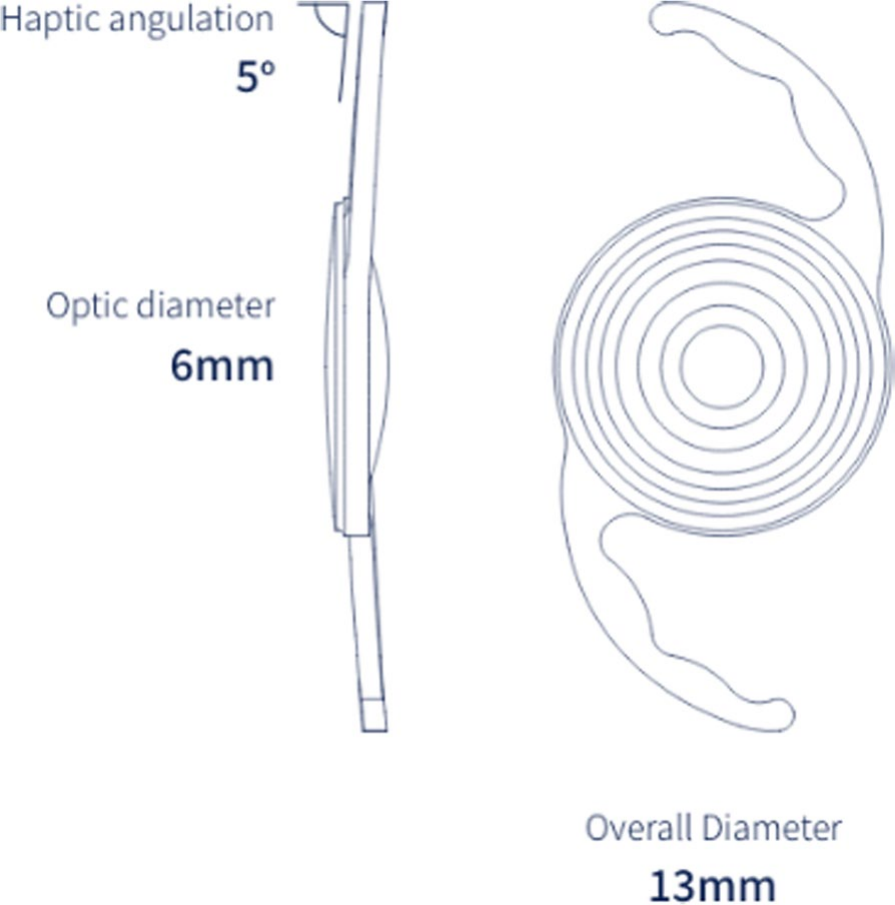
The Intensity multifocal IOL

The Intensity SL is a foldable, single-piece IOL with C-loop haptics and an angulation of 5 degrees. It is made of hydrophilic acrylic with 25% water content and has both an ultraviolet light blocker and a natural yellow violet light filter (Fig. 1).

The IOL profile consists of three zones, each of which (according to the manufacturer’s product information) is optimized by the Dynamic Light Utilization algorithm. The resulting multiple zones are designed to provide good visual quality for different pupil sizes and all lighting conditions. Based on this algorithm, the modulated transfer function (MTF) is increased in two areas: between far and intermediate and between intermediate and near, allowing for a continuous defocus curve. The lens is therefore essentially a 5-focus design with these two additional foci (or intensifiers) in addition to the far, intermediate and near foci that are part of the optical design of most existing multifocal IOLs. The addition of the two smaller peaks is intended to provide a smoother transition across distances than current multifocal IOLs with three foci. Laboratory evaluation has documented a light energy utilization of 93.5%, which is higher than some other multifocal lenses that result in a loss of only about 6.5% of light energy.

The total diameter of the Intensity SL is 13 mm, the diameter of the optical zone is 6 mm and the angulation is 5 degrees. The lens has directionality: the leading haptic must point left; the IOL is dialed clockwise. The lens is designed for implantation in the capsular bag.

### Surgical technique

All surgeries were performed by one experienced surgeon (G.G.). In all cases, a standard small-incision cataract extraction was performed with a clear corneal incision of 2.2 to 2.8 mm. The anterior capsulotomy was performed using a continuous curvilinear capsulorhexis with an intended diameter between 5.0 and 5.5 mm. If deemed necessary by the surgeon, a capsular tension ring could be placed during the next steps of the procedure. Phacoemulsification was used for lens fragmentation, and the fragmented lens was removed by irrigation/aspiration. Through the incision, the folded IOL was placed in its cartridge into the capsular bag. The refractive goal was emmetropia in all cases. At the end of the procedure an antibiotic (cefuroxime) was injected into the anterior chamber and all traces of viscoelastic were removed. The corneal incision was closed with corneal stromal hydration, no sutures were required. Postoperatively, both topical antibiotic (gatifloxacin) and topical steroid (prednisolone) were applied.

## Results

A total of 31 patients underwent implantation of an Intensity IOL multifocal lens; 30 patients underwent bilateral implantation and one patient underwent monolateral implantation; a total of 61 eyes were included in this study. The mean age of the patients was 50 years. None of the patients dropped out of the protocol during the 3-month follow-up.

**Fig. 2:**
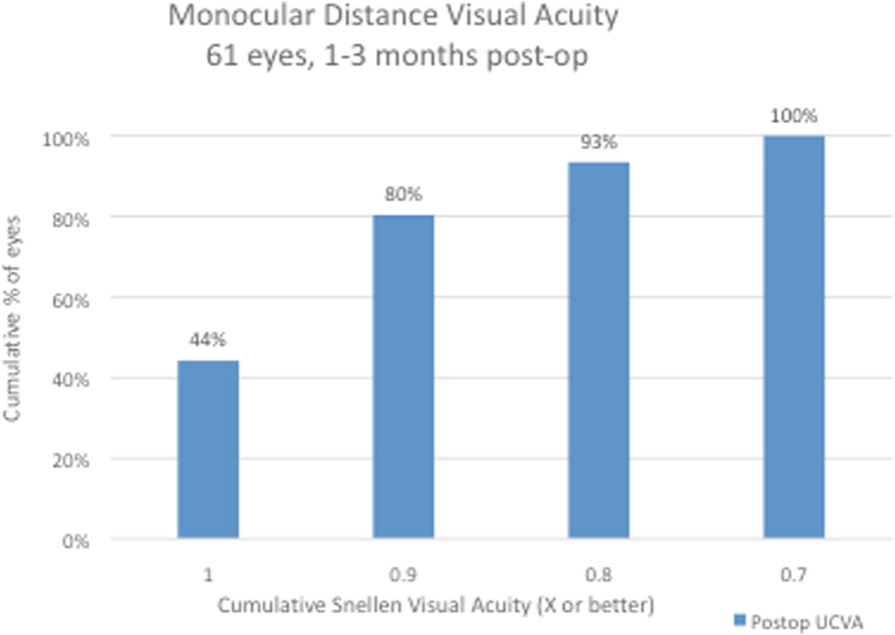
Monocular distance uncorrected visual acuity (UDVA). All 61 eyes (100%) had an uncorrected distance visual acuity of at least 0.7; 57 eyes (93%) had a UCDVA of 0.8 [0.1 logMAR] and 27 eyes (44%) had a UCDVA of 1.0 [0.0 logMAR] with a mean UCDVA of 0.93 [0.04 logMAR]

**Fig. 3:**
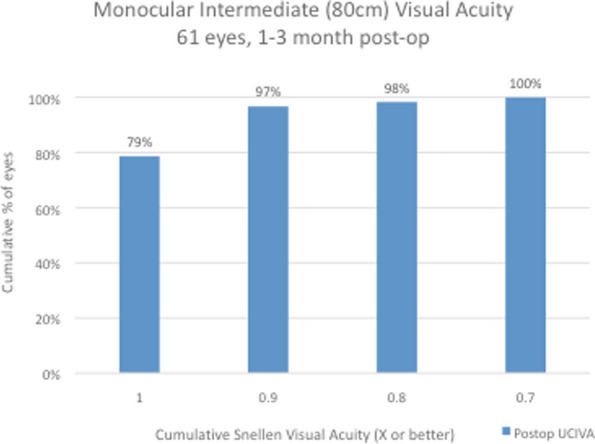
Monocular intermediate (80 cm) uncorrected visual acuity (UIVA): 60 eyes (98%) had a UCIVA of at least 0.8 [0.1 logMAR] and 48 eyes (79%) had a UCIVA of 1.0 [0.0 logMAR] at 80 cm distance, with a mean uncorrected monocular intermediate vision of 0.97 [0.02 logMAR] in the 61 eyes and 1.0 [0.0 logMAR] when tested binocularly

**Fig. 4:**
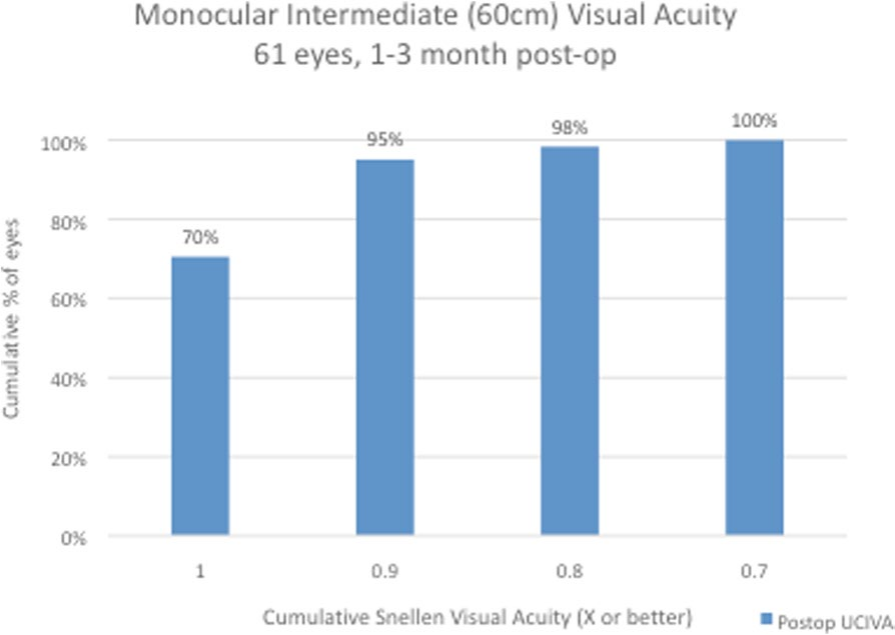
Monocular intermediate (60 cm) uncorrected visual acuity (UIVA): At 60 cm, 98% (*n* = 60) of the operated eyes had an UCIVA of at least 0.8 [0.1 logMAR] at this shorter intermediate distance, with all of them having a UCIVA of at least 0.7 [0.15 logMAR] and an average of 0.96 (monocular) [0.02 logMAR] and 0.98 (binocular) [0.01 logMAR]

**Fig. 5:**
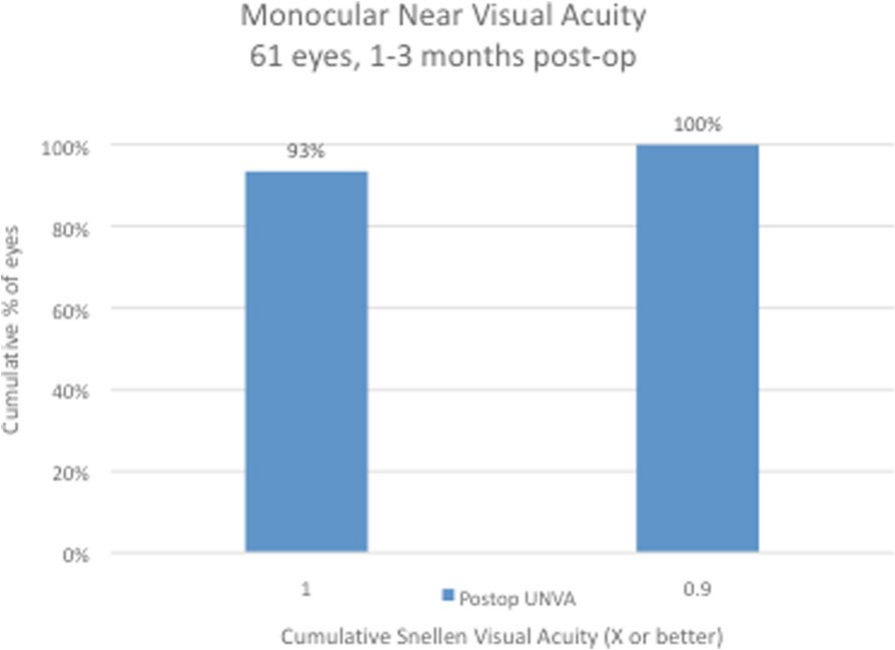
Monocular near uncorrected visual acuity (UDVA): For reading distance, all eyes had uncorrected near acuity of 0.9 [0.05 logMAR] and 57 eyes (93%) had UCNVA of 1.0 [0.0 logMAR] with an average binocular DCNVA of 1.02 [-0.02 logMAR]

Snellen visual acuity results at 1 to 3 months after surgery are shown in Figs. 2, 3, 4 and 5 and Table 1. All 61 eyes (100%) had an uncorrected distance visual acuity of at least 0.7; 57 eyes (93%) had a UCDVA of 0.8 [0.1 logMAR] and 27 eyes (44%) had a UCDVA of 1.0 [0.0 logMAR] with a mean UCDVA of 0.93 [0.04 logMAR] (Fig. 2). When testing binocular visual acuity, all patients had a BCDVA of 0.9 [0.05 logMAR] and 26 patients (83%) had a BCDVA of 1.0 [0.0 logMAR]. When assessing intermediate vision, 60 eyes (98%) had a UCIVA of at least 0.8 [0.1 logMAR] and 48 eyes (79%) had a UCIVA of 1.0 [0.0 logMAR] at 80 cm distance, with a mean uncorrected monocular intermediate vision of 0.97 [0.02 logMAR] in the 61 eyes and 1.0 [0.0 logMAR] when tested binocularly (Fig. 3). The results for intermediate vision at 60 cm were almost identical: 98% (*n* = 60) of the operated eyes saw at least 0.8 [0.1 logMAR] at this shorter intermediate distance, with all of them having a UCIVA of at least 0.7 [0.15 logMAR] and an average of 0.96 (monocular) [0.02 logMAR] and 0.98 (binocular) [0.01 logMAR] (Fig. 4). For reading distance, all eyes had uncorrected near acuity of 0.9 [0.05 logMAR] and 57 eyes (93%) had UCNVA of 1.0 [0.0 logMAR] with an average binocular DCNVA of 1.02 [-0.02 logMAR] (Fig. 5).

**Table 1:** Average monocular uncorrected and binocular distance-corrected visual acuities 3 months postoperatively in Snellen

**Averages:**		
**Monocular uncorrected measurements:**	**n**	**average**
Monocular UCVA	61	0.93 ± 0.11
Monocular UIVA at 80 cm	61	0.97 ± 0.06
Monocular UIVA at 60 cm	61	0.96 ± 0.06
Monocular UNVA	61	1 ± 0.05
**Binocular distance‐corr. measurements:**	**n**	**average**
Binocular CDVA	30	1 ± 0.07
Binocular DCIVA at 80 cm	30	1 ± 0.08
Binocular DCIVA at 60 cm	30	0.98 ± 0.1
Binocular DCNVA	30	1.02 ± 0.07

Visual phenomena were not reported by 20 of the 31 patients; 4 patients described them as “mild” when specifically asked, and 7 patients observed halos, glare, and similar visual disturbances.

The safety profile of the Intensity SL appeared to be excellent, with none of the 61 eyes losing one or more lines of BCVA on the Snellen charts. There were no significant post-operative complications. At 3 months, there was no visually relevant posterior capsular opacification (PCO) or macular edema in this patient population. The IOL was well centered in all eyes and there was no dislocation or tilt. All IOP measurements were within normal limits, endothelial cell loss was moderate.

## Discussion

Cataract surgery today is almost always, as the late Emmanuel Rosen rightly postulated, refractive surgery [6] with the potential to treat refractive errors with a high degree of effectiveness [7] and to provide patients with good vision at various distances without the need for spectacles. This is especially true for the one refractive error that the eye doctor diagnoses in the vast majority of cataract patients: Presbyopia is one of the most common refractive problems, currently affecting approximately 1.8 billion people worldwide and expected to increase to 2.1 billion by 2030 [8]; uncorrected or undercorrected presbyopia is estimated to result in annual global productivity losses of approximately $25 million [9].

Given this high demand for presbyopia correction, if possible at the time of cataract surgery—which is always an important event in an individual’s life—there is a need for new IOLs that can provide better vision at different distances than earlier generations of lenses. This is especially true for the intermediate distance which plays an important role in the daily lives of today’s seniors. The data from this limited “real-world” study support the view that the Intensity SL has the potential to provide patients with good visual acuity and to contribute positively to their vision-related quality of life.

We have described clinical results with this new multifocal IOL whose optical design promises to smooth the transitions between the traditional 3 foci (distance, intermediate, near) of diffractive multifocal lenses on the market. The results presented here are favorable, with virtually all patients achieving acceptable or even excellent uncorrected visual acuity at the different distances tested (distance as well as near and intermediate vision at two different distances, arm’s length and slightly more than arm’s length). The one patient with a unilateral implant was informed of the pros and cons of unilateral surgery. She understood and insisted on the procedure in light of her work-related vision needs as a journalist. After surgery, the patient was satisfied with the results.

As this is a novel design, to our knowledge only two other peer-reviewed studies with comparable patient populations have been published. Our results support both of their findings. Bianchi implanted the Intensity IOL in 112 eyes of 56 patients. None of them experienced visual loss and 94% had a final refraction within ± 0.5 D; uncorrected and corrected visual acuity in this group did not differ much from our results [10]. The group of Ehud Assia et al. implanted the Intensity SL in 20 patients (40 eyes) and reported a mean uncorrected visual acuity for distance, intermediate and near of 0.03, 0.09 and -0.22 logMAR, respectively; the excellent result for near seems to reflect our experience. The authors also report a high level of patient satisfaction [11].

Our study has limitations. The patient population is rather small and the follow-up is only 3 months. Larger studies with longer follow-up are needed to support the promising clinical results we can present here. Also, we only had patients with the Intensity SL; this is not a comparison with another multifocal IOL based on a different design, which would be very interesting. Longer follow-up would also be valuable to assess the degree of visual disturbance (although relatively low in our population), as it is hypothesized that neuroadaptation will reduce or even eliminate these symptoms over time.

Possible directions of future research would be a) a long-term follow-up (2 to 4 years postoperatively) of the same patients operated with Intensity SL and b) a comparative clinical trial, with Intensity SL vs. an equivalent IOL. The latter could, for instance, be the Panoptix Model TFNT00 (Alcon). It has a quadrifocal design, although it is widely described as a trifocal intraocular lens. According to a recent publication in BMC Ophthalmology, this IOL consists of a large 4.5 mm diffractive zone and 15 diffractive zones and an outer refractive edge. There are three focal points from distance to intermediate and near, splitting incident light to produce mid- and near-range diopters of 2.17 diopters (D) and 3.25 D, respectively. As Qu et al. point out, the Panoptix TFNT00 offers the best reading distances of 60 cm and 42 cm. This novel diffractive structure is said to provide high light utilization, delivering 88% of the light that simulates a 3.0 mm pupil size to the retina. This light energy is split 25% for nearsightedness and intermediate vision, and 50% for farsightedness. Qu et al. reported good visual results up to a near distance of 33 cm, although visual acuity at this short distance was not as good as visual acuity at 40 cm [12]. In a cohort of 40 patients (80 eyes), Jo et al. documented mean uncorrected visual acuities for distance, intermediate and near of 0.04, 0.04 and 0.03 logMAR, respectively. At all distances, high spectacle independence was observed, with 37.5% of patients reporting photic phenomena [13]. In a larger Japanese study by Kawamura et al. of 122 eyes implanted with this new IOL compared to 1326 eyes with a diffractive bifocal intraocular lens, uncorrected intermediate visual acuity, contrast sensitivity and contrast sensitivity with glare were significantly better in the Panoptix group [14].

## Conclusions

The new Intensity SL multifocal IOL produced very favorable visual results at a variety of distances, with good results at intermediate distances (and thus for using electronic devices such as tablets, computers and smart phones), and caused visual phenomena in a minority of patients. This five-focus lens appears to have a good safety profile. The majority of patients did not experience or were only mildly bothered by optical phenomena such as halos and glare.

### Supplementary Information

Supplementary Material 1.

## Data Availability

Datasets have been submitted. The datasets used and/or analyzed in the current study are available from the corresponding author upon reasonable request.

## Abbreviations

IOL: Intraocular lens
EDOF: Extended depth of focus
UCVA: Uncorrected visual acuities
UCDVA: Uncorrected distance visual acuity
UCIVA: Uncorrected intermediate visual acuity
UCNVA: Uncorrected near visual acuity
BCVA: Best corrected visual acuities
BCDVA: Best corrected distance visual acuity
BCIVA: Best corrected intermediate visual acuity
BCNVA: Best corrected near visual acuity
IOP: Intraocular pressure
ECD: Endothelial cell density
D: Diopters
MTF: Moduled transfer function
PCO: Posterior capsular opacification
FLACS: Femtosecond laser-assisted cataract surgery
